# The effect of dance-based mind-motor activities on the quality of life in the patients recovering from COVID-19: A protocol for systematic review and meta-analysis

**DOI:** 10.1097/MD.0000000000025102

**Published:** 2021-03-19

**Authors:** Yi Ding, Chenchen Guo, Shaohong Yu, Peng Zhang, Ziyun Feng, Jinglong Sun, Xiangxia Meng, Li Li, He Zhuang

**Affiliations:** a The Second Affiliated Hospital of Shandong University of Traditional Chinese Medicine; b Neck-Shoulder and Lumbocrural Pain Hospital Affiliated to Shandong First Medical University, Jinan; c The Second Clinical Medical College of Shandong University of Traditional Chinese Medicine; d Rizhao Hospital of Traditional Chinese Medicine; e School of Rehabilitation Medicine, Shandong University of Traditional Chinese Medicine, Jinan, Shandong, China.

**Keywords:** coronavirus disease 2019, dance-based mind-motor activities, protocol, systematic review

## Abstract

**Background:**

Since the outbreak of coronavirus disease 2019 (COVID-19), with the improvement of diagnosis and treatment level in various countries, more and more patients have been discharged after systematic treatment. In order to effectively promote the overall recovery of patients’ physical and mental function and quality of life (QOL), the focus of clinical work should be gradually shifted to rehabilitation treatment. Dance-based mind-motor activities were defined as coordinated upright mind-motor movements that emphasize dynamic balance, structured through music or an inner rhythm (e.g., breathing) and distinctive instructions or choreography, and that involve social interaction. It has positive effects on motor function, lung function, psychological mood and other aspects, so it can be used as a safe alternative therapy for patients recovering from COVID-19. At present, there are no relevant articles for systematic review.

**Methods:**

From its inception until March 2021, we will conduct a comprehensive electronic search, including Cochrane Library, MEDLINE, PubMed, Springer, EMBASE, Chinese Science Citation Database, China National Knowledge Infrastructure, Chinese Biomedical Literature Database, Chinese Scientific Journal Database, Wan-fang database. Two independent researchers will conduct article retrieval, screening, quality assessment, and data analysis through the Review Manager (V. 5.3.5).

**Results:**

The results of this study will evaluate the effectiveness and safety of dance-based mind-motor activities for the improvement of QOL in COVID-19 patients during the recovery period.

**Conclusion:**

The conclusion of the study will provide an evidence to judge whether dance-based mind-motor activities is effective and safe for COVID-19 in recovery period.

**Ethics and dissemination:**

This protocol will not evaluate individual patient information or infringe patient rights and therefore does not require ethical approval.

**PROSPERO registration number:**

CRD42021232995.

## 1 Introduction

Coronavirus disease 2019 (COVID-19) was found to be caused by a novel viral pathogen named as SARS-CoV-2. COVID-19 first appeared in Wuhan, China, in early 2019, then broke out in China, and was further declared a pandemic due to its rapid and indiscriminate spread in different parts of the world.^[1,2]^ So far, more than 102,942,987 people have been confirmed cases of COVID-19, including 2,232,233 deaths.^[3]^ Patients had several common symptoms at onset of COVID-19 illness are fever, cough, and fatigue, while other symptoms include sputum production, headache, hemoptysis, diarrhoea, dyspnoea, lymphopenia, and radiographic indication of pneumonia.^[4–7]^ The clinical manifestation of COVID-19 can vary from asymptomatic and mild flu-like symptoms to acute respiratory distress syndrome and death. There is no validated treatment for COVID-19 currently. Current treatments are mainly symptomatic supportive care, which helps patients gradually recover from the disease.

An increasing number of studies have found that COVID-19 can lead to long-term lung, heart, kidney, and nervous system complications and may have a greater impact on QOL.^[8–10]^ In addition, prolonged hospitalization or bedridden treatment in COVID-19 patients may result in motor incapacity, which can lead to increased pain and decreased motor function.^[11]^ The COVID-19 pandemic has had a worrying impact on emotional and social functioning among patients, often leading to mental health problems such as anxiety, depression and insomnia.^[12]^ To sum up, COVID-19 will reduce the body function, reduce the exercise ability and have an unhealthy impact on the psychological health, and thus adversely affect the QOL of patients. However, there is a lack of studies on the improvement of QOL in patients with COVID-19 sequelae stage, and there is an urgent need for a simple, reliable and feasible treatment to improve QOL.

As more and more COVID-19 patients are cured and discharged, the focus of clinical work should be gradually shifted to the improvement of related sequelae and the improvement of QOL. However, there is a lack of studies on the improvement of QOL of patients with COVID-19 sequelas at present, and there is an urgent need for an operable, simple, safe and effective treatment that is free from space constraints and conducive to promotion to help patients recover in the later stage and improve their QOL.^[13]^

Dance-based mind-motor activities can be performed solo, in a pair, or in group formations, without of regional restrictions. In addition to several styles of well-known dance-based mind-motor activities, such as folk or ballroom dancing, Tai chi, Baduanjin, Qigong, Yijinjing, and Daoyin fulfills the above definition. Contrarily, static exercises such as Yoga and Liuzijue are excluded because they are not dynamic exercises and lack of social activities. Dance-based mind-motor activities have been suggested as physical exercise^[14–16]^ with extended benefits beyond the physical on cognition,^[17,18]^ social interaction,^[19,20]^ QOL,^[21]^ and motivation to be physically active.^[22,23]^ It has the characteristics of good analgesic effect, improve exercise ability, no side effects, convenient operation, low economic burden, and more conducive to promoting the rehabilitation of patients.

At present, there is still a lack of evidence-based medicine in the treatment of COVID-19 patients in rehabilitation, and it is very necessary to improve anxiety, tension, and other negative emotions of patients in rehabilitation and improve their QOL. Therefore, it is necessary to review it and provide evidence for clinicians.

## 2 Methods

This study protocol has been registered on PROSPERO register of systematic review (No. CRD42021232995). The procedure of this protocol will be based on the Cochrane Handbook (5.2.0) as preferred guidance.

### 2.1 Inclusion criteria for study selection

#### 2.1.1 Types of studies

As the randomized controlled trials (RCTs) are reliable and feasible, RCTs will be included only, regardless of whether blind method and allocation concealment are used. Non-RCT, quasi-RCT, and any other type of study will be excluded.

#### 2.1.2 Types of patients

Patients with COVID-19 in recovery period, regardless of race, sex, nationality, economic status, education, or medical unit.

#### 2.1.3 Type of interventions

Dance-based mind-motor activities include: Tai chi, Baduanjin, Qigong, Yijinjing, Daoyin, Tango dance program etc. We will compare the following interventions: treatments other than dance-based mind-motor activities (e.g., routine health guidance, drug treatments, breathing training, etc.).

#### 2.1.4 Type of outcome measure

##### 2.1.4.1 Primary outcomes

1. Pulmonary function test, including:
  1. Forced vital capacity (FVC);
  2. Forced expiratory volume in 1 second/Forced vital capacity (FEV1/FVC);
  3. Forced expiratory volume in 1 second/prediction (FEV1/PRE);
  4. Forced expiratory volume in 1 second (FEV1).
2. 6-Minute walk test (6MWT);
3. Pulse oxygen saturation (SpO_2_);
4. TCM efficacy criteria.

##### 2.1.4.2 Secondary outcomes

1. Self-rating anxiety scale (SAS);
2. Self-rating depression scale (SDS);
3. Quality of life (QOL);
4. Short Form-36 (SF-36);
5. Pittsburgh Sleep Quality Index (PSQI);
6. Hamilton Depression Scale (HAMD);
7. Adverse events.

### 2.2 Data collection and analysis

#### 2.2.1 Search strategy

From the beginning to February 2021, we will retrieve the following electronic databases: Cochrane Library, MEDLINE, PubMed, Springer, EMBASE, CSCD, CNKI, CBM, VIP, Wan-fang database. In addition, we also plan to do a manual search on Google academic. The language is limited to English and Chinese. The search terms are shown in Table 1.

**Table 1:** Search strategy for PubMed database.

Number	Strategy
#1	“Tai chi” OR “Tai ji” OR “Tai chi” OR “Tai chi chuan” OR “Tai ji” OR “Tai ji quan” OR “Tai ji quan” OR “martial arts” OR “shadowbox” OR “Baduanjin” OR “Yijinjing” OR “Qigong” OR “Chi Kung” OR “DAOYIN”
#2	“folk dancing” OR “ballroom dancing” OR “Chinese Square dancing” OR “Low-impact aerobic dance” OR “Tango dance program”
#3	#1 OR #2
#4	“COVID-19” OR “2019 novel coronavirus infection” OR “2019-nCoV infection” OR “COVID-19 pandemic” OR “coronavirus disease-19” OR “2019-nCoV disease” OR “COVID19” OR “2019 novel coronavirus disease” OR “coronavirus disease 2019” OR “coronavirus” OR “SARS-CoV-2 infection”
#5	“Randomized controlled trials” OR “Randomized” OR “Randomly” OR “Clinical trial” OR “Controlled clinical trial” OR “Double-blind method” OR “Single-blind method”
#6	#3 AND #4 AND #5

#### 2.2.2 Study selection

**Figure 1:**
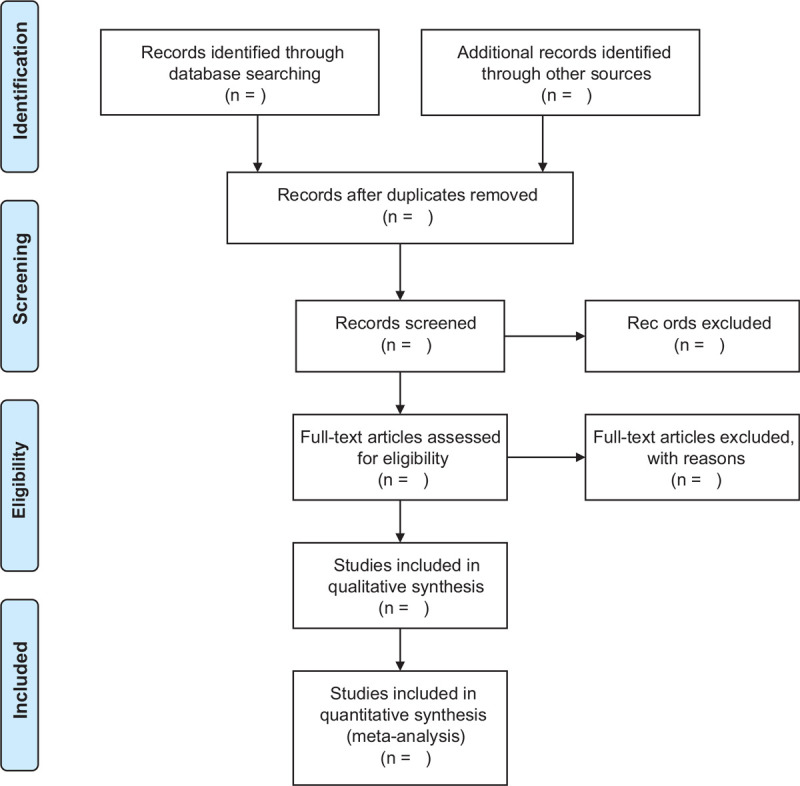
Flow diagram of literature retrieval.

First of all, all references will be input into EndNote (X9), and all duplicate references will be deleted. Second, 2 independent researches (YD and CG) will read the title, abstract, and full text, respectively, based on the inclusion criteria, to determine which clinical trials to include. When complete literature or necessary data is not available, we will attempt to contact the appropriate authors. If there is any disagreement, a third party experts (HZ) will be consulted for resolution. Figure 1 shows the process of literature screening.

#### 2.2.3 Data extraction and management

Two independent authors (PZ and ZF) extracted the following data using a data record table prepared:

1. Journal, the information about the author, title, the time of publication, Country and region;
2. The characteristics of participants, sample size;
3. Interventions: Dance-based mind-motor activities styles, Training frequencies and training time of every time, Total training time;
4. Study design: Randomization, Blinding, Result analysis;
5. Primary and secondary outcome measurements as well as any adverse events.

All of the above data and information will be managed using Microsoft Excel 2019.

#### 2.2.4 Assessment of risk of bias in included studies

Two researchers (JS and SY) will use Cochrane Handbook (5.2.0) tool to independently evaluate the risk of bias in the included studies. We will evaluate the article included 6 items: (a) random sequence generation; (b) allocation concealment; (c)blinding methods; (d) completeness of outcome data; (e) selective reporting; (f) other sources of bias. They are divided into 3 different levels: Blur, Low, and High based on the included literature in the above evaluation items. If the information is incomplete, we will contact the first author of the article. In case of disagreement between the 2 researchers, discussion will be held with the third expert (HZ).

#### 2.2.5 Measures of treatment effect

In this protocol, 2 independent researches (YD and LL) will use 95% confidence interval risk ratio to rigorously analyze the dichotomous data. And for the continuous data, mean difference or standard median difference is used to measure the efficacy of 95% confidence interval.

#### 2.2.6 Dealing with missing data

If the included RCT literature data information is incomplete, we will contact the first author by making a phone call or sending emails. If the required data is still not available, the literature will be deleted.

#### 2.2.7 Assessment of heterogeneity

The heterogeneity of the data will be analyzed by Q-test and *I*^2^ statistic. The heterogeneity will be deemed as low when *I*^*2*^ < 50%, moderate (50%–75%), high (*I*^*2*^ > 75%). It is generally believed that *I*^*2*^ ≥ 50% indicates substantial heterogeneity.

#### 2.2.8 Assessment of reporting bias

Funnel plots will be established to evaluate the reporting biases, when there are over 10 RCTs included in the meta-analysis.

#### 2.2.9 Data synthesis

RevMan V.5.3.5 will be used for data synthesis. Fixed-effect model with merger analysis will be applied when heterogeneity is low. When the heterogeneity is moderate, the random-effect model is used for merger analysis. While the heterogeneity is significantly high, descriptive analysis will be performed.

#### 2.2.10 Subgroup analysis

Subgroup analysis will be conducted if data are available. Factors such as different types of control interventions (e.g., Tai chi, Baduanjin, Yijinjing, Daoyin, Tango dance program etc.) and different outcomes will be considered.

#### 2.2.11 Grading of evidence quality

We will use the Grading of Recommendations Assessment approach for the assessment of the strength of evidence for each outcome. The result will be classified as high, moderate, low, or very low.

#### 2.2.12 Ethics and dissemination

This protocol will not evaluate individual patient information or infringe patient rights and therefore does not require ethical approval.

## 3 Discussion

With the effective intervention of targeted therapy, more and more patients with COVID-19 are cured and discharged from hospital, and a large number of patients will enter the recovery stage. At 6 months after symptom onset, such as impaired lung function, fatigue, muscle weakness, sleep difficulties, anxiety, and depression were the main symptoms of patients who had recovered from COVID-19.^[24]^ Evidence supports that dance-based mind-motor activities can safely and effectively improve patients’ fatigue, reduced exercise ability, impaired lung function and other problems, as well as significantly improve patients’ anxiety, depression, insomnia and other states.^[25–31]^

However, there are no systematic review of the effects of dance-based mind-motor activities on QOL in convalescent patients with COVID-19. This system review will be the first to evaluate the efficacy and safety of dance-based mind-motor activities in improving the QOL of convalescent patients with COVID-19. This review can provide a decision-making basis and clinical reference for clinicians to choose alternative therapy to improve the QOL of patients who had recovered from COVID-19. Nonetheless, the lack of sufficient and high-quality RCTs may be a limitation for this meta-analysis.

## Author contributions

**Conceptualization:** Yi Ding, Li Li.

**Data curation:** Chenchen Guo, Peng Zhang, Xiangxia Meng.

**Investigation:** Shaohong YU.

**Methodology:** Yi ding, Chenchen Guo, Jinglong Sun.

**Validation:** Ziyun Feng, He Zhuang.

**Visualization:** Peng Zhang.

**Writing – original draft:** Yi Ding, Chenchen Guo.

**Writing – review & editing:** Jinglong Sun.
